# Millimeter-Wave Radar Localization Using Indoor Multipath Effect

**DOI:** 10.3390/s22155671

**Published:** 2022-07-29

**Authors:** Zhanjun Hao, Hao Yan, Xiaochao Dang, Zhongyu Ma, Peng Jin, Wenze Ke

**Affiliations:** College of Computer Science & Engineering, Northwest Normal University, Lanzhou 730070, China; 2020222038@nwnu.edu.cn (H.Y.); dangxc@nwnu.edu.cn (X.D.); mazybg@nwnu.edu.cn (Z.M.); 2021222246@nwnu.edu.cn (P.J.); 2021222210@nwnu.edu.cn (W.K.)

**Keywords:** wireless sensing, millimeter-wave, multipath exploitation, indoor location

## Abstract

The positioning of indoor electronic devices is an essential part of human–computer interaction, and the accuracy of positioning affects the level of user experience. Most existing methods for RF-based device localization choose to ignore or remove the impact of multipath effects. However, exploiting the multipath effect caused by the complex indoor environment helps to improve the model’s localization accuracy. In response to this question, this paper proposes a multipath-assisted localization (MAL) model based on millimeter-wave radar to achieve the localization of indoor electronic devices. The model fully considers the help of the multipath effect when describing the characteristics of the reflected signal and precisely locates the target position by using the MAL area formed by the reflected signal. At the same time, for the situation where the radar in the traditional Single-Input Single-Output (SISO) mode cannot obtain the 3D spatial position information of the target, the advantage of the MAL model is that the 3D information of the target can be obtained after the mining process of the multipath effect. Furthermore, based on the original hardware, it can achieve a breakthrough in angular resolution. Experiments show that our proposed MAL model enables the millimeter-wave multipath positioning model to achieve a 3D positioning error within 15 cm.

## 1. Introduction

With the development of electronic information technology, Location-Based Services (LBS) are closely involved in people’s lives [1,2]. The location information of small electronic devices and sensors is essential for human–computer interaction. The accurate location information of devices can give users a strong sense of experience—for example, in Virtual Reality (VR) [3,4] and indoor monitoring. With the development of sensing technologies, the indoor positioning tasks of electronic devices can be achieved using popular wireless infrastructures, such as commercial Wi-Fi devices, Bluetooth devices, Ultra Wide Band (UWB) devices, Radio Frequency Identification (RFID) devices, cameras, and millimeter-wave radar. These wireless devices can obtain the target device’s location in physical space.

Currently, the positioning of outdoor devices mainly relies on BeiDou Navigation Satellite System (BDS) or Global Positioning System (GPS) satellite signals [5] and Global System for Mobile Communications (GSM) base stations [6]. The two positioning methods are similar in principle and determine the location of the device based on the distance between different satellites or base stations to the receiver, with accuracy at the meter level. Indoor positioning relies on signals from Bluetooth or Wi-Fi [7,8]. The Bluetooth-based and Wi-Fi-based positioning methods require feedback from the device and have an error of approximately 1 m [8]. The UWB positioning system [9], which achieves centimeter-level positioning accuracy, requires the installation and deployment of several specialized hardware systems, and the system deployment cost is high. The number of its base stations limits the accuracy. Inexpensive RFID positioning systems are limited by their sensing distance. In recent years, with the development of computer vision, camera-based localization techniques have also achieved good localization results [10,11,12]. However, they face privacy issues in private environments, the effect of light, and the inability to localize in non-line-of-sight (NLOS) environments.

Radar uses changes in the phase of electromagnetic wave signals to detect and range targets. In its early days, it was used in military applications such as missile guidance and battlefield surveillance. However, with the civilization of radar technology and the rapid development of chip and antenna technology, researchers are paying more and more attention to millimeter-wave radar. Compared with RF devices such as UWB, WIFI, and RFID, millimeter-wave radar has the advantages of short wavelength, high bandwidth, and high directionality. These advantages determine that the limits of millimeter-wave radar sensing are much larger than other sensing devices.

The phase change is the hub that connects the real world to the computer world and is the “bridge” for wireless sensing by millimeter-wave radar. By analyzing the phase changes in the received signal, researchers can obtain information about the target’s distance, angle, and speed. It is increasingly used as an efficient sensing tool in many scenarios [13,14]. Since, in most cases, multipath effects impact the electromagnetic wave perception of the physical world, many approaches choose to ignore or remove the complex multipath effects in indoor environments. However, when using proper modeling, multipath effects have their advantages [15,16].

This work proposes a model for the high-precision localization of millimeter-wave radar adapted to multipath effects in different environments to address the above problems. The model calculates the radar sensing area in the environment by describing the relationship between signals in the multipath effect and achieves the 3D high-precision localization of indoor devices. Our contributions are as follows:

1. A perception model based on the multipath effect is proposed to solve the localization problem of indoor electronic devices. The model addresses the indoor high-precision localization problem by mining multipath information and using the target radial distance instead of the target Angle-of-Arrival (AoA).
2. A model for extracting micro-Doppler at a specific distance is proposed for achieving dynamic object capture. The model solves the micro-Doppler feature-to-distance mapping by superimposing the Doppler information at a specific distance, which enhances the feature information on the distance plane of the object.
3. The rule of constant radar 2D perceived area and perceived area on the same distance plane based on the multi-order reflection model is proposed. Based on this rule, the three-dimensional positioning problem of horizontally aligned antennas and the angular resolution problem in the case of insufficient antenna arrays are solved.

The rest of the paper is organized as follows: Section 2 discusses the work related to indoor localization. Section 3 describes the MAL model. In Section 4, we describe the experimental design and evaluate the results. We conclude the entire paper and discuss future work in Section 5.

## 2. Related Work

In this section, we discuss the literature on millimeter-wave radar and the principles of millimeter-wave radar indoor localization technology.

### 2.1. Millimeter-Wave Radar Applications

Researchers have used millimeter-wave radar for the high-resolution imaging and recognition of objects [17,18] by continuously moving the radar to achieve the effect of simulating an antenna array and constructing a two-dimensional and three-dimensional holographic projection of the object with both longitudinal and lateral accuracy in the millimeter range. In [19,20,21,22], the authors use synthetic aperture radar (SAR) combined with a deep learning approach to achieve high-resolution imaging of ship resources to observe and manage ship resources. Furthermore, compared with the traditional feature extraction method, the authors have greatly improved the ship detection accuracy and speed by optimizing the network structure [23,24,25]. Millimeter-wave radar is sensitive to vibrating objects, and in [26], the authors used millimeter-wave radar to capture chest vibrations for non-contact vital sign detection. In [27], Junchen Guo et al. used the captured mechanical vibrations to determine whether there are abnormalities in the operation of machines and equipment.

Researchers have also obtained target micro-Doppler features by superimposing target velocity information from multiple Chirp radar signals to capture micro-motion features caused by rigid or non-rigid body motion [28,29]. In a recent study, Kim et al. [30] used deep learning methods to achieve 97.6% and 90.3% accuracy for human detection and human classification activities. In [29], the authors collected gait information from volunteers walking freely in an indoor environment by radar and used the same deep learning approach for classification. The purpose of identifying people is achieved by classifying different gait information. In [31], Saho K et al. used the micro-Doppler effect of radar to analyze the gait of individuals to study the gait differences related to fall risk and detect individuals with a higher risk of falling. However, most researchers have ignored the use of micro-Doppler for localization. This paper locates the position of the target by judging the upper micro-Doppler features of different distance surfaces.

Millimeter-wave radar still performs well in LBS. In outdoor environments, researchers have used radar to locate the rough location of people, whether adults or children, even at 100–150 m [32]. Indoors, the authors introduced the first RF signal-based RF-Pose3D system that uses millimeter-wave radar to construct the human 3D skeleton. RF-Pose3D uses a CNN for high-dimensional convolutional decomposition into lower dimensions to compress the RF signal’s spatio-temporal information and track the skeleton of each part of the volunteer’s body with a tracking error at centimeter level [33]. In [34], the author used backscattered signals to track the body position using a neural network approach. An accuracy of 12 cm was achieved in the single-person case and 21 cm in the multiple-person case. In [35], the WiTrack system was able to provide quality 3D human motion tracking with an average error of less than 12 cm in all cases.

Michael Leigsnering et al. introduced a compression-aware approach for the joint localization and velocity estimation of targets in indoor multipath environments by analyzing multipath signals [36]. Similarly, Pawan Setlur et al. enhanced the detection and localization accuracy of targets behind walls with the help of the multipath effect [37].

In [32,33,34,35], the authors ignore the help of multipath effects. Meanwhile, in [36,37], the authors exploited the multipath effect but ignored the improvement in the multipath effect regarding the longitudinal angular resolution of the radar. Based on the above problems, we propose a model that takes advantage of the high-bandwidth property of reflected signals to solve the problem of the 3D localization of horizontally aligned antennas and how to distinguish multiple targets with different AOA at the same distance in SISO mode.

### 2.2. Principles of Radar Perception

For millimeter-wave radar-based wireless sensing, by observing the phase difference of the received signal, we can make a series of estimates of the target displacement, velocity, and angle. For a single stationary object, the radar generates a frequency-invariant cosine signal, and multiple cosine signals by superposition constitute a radar Intermediate Frequency (IF) signal in Equation (1), which contains information on the distance, velocity, and angle of the object:

(1) $$S\left(t\right)=Le^{j4\pi \left(f_{c}+Kt\right)R\left(t\right)/c}$$

In Equation (1), $f_{c}$ is the initial radar transmitting antenna RF frequency, and R(t) denotes the distance information. *K* is the ratio of the bandwidth *B* to the duration $T_{c}$ of a single Frequency Modulated Continuous Wave (FMCW) signal, and *L* is the loss in the path.

After the Range-FFT, the distance information of the objects in space can be obtained. The specific procedure is as follows, in Equations (2) and (3):

(2) $$\begin{array}{c} S^{\left[n\right]}\left(t\right)\overset{h(m)\&Range-FFT}{\to }S_{range}^{\left[n\right]}\left(t\right)=L^{\left[n\right]}e^{R_{rangle}^{\left[n\right]}\left(t\right)\left[\frac{4j\pi (f_{c})}{c}\right]} \end{array}$$

In this paper, the target distance is sensed by FFT operation, so the distance resolution is ΔR, ($\Delta R_{\max}=c/\left(2B_{\max}\right)\approx 4$ cm). Moreover, the distance represented by the range bin is a discrete sequence.

(3) $$\begin{array}{c} S_{range}^{\left[n\right]}\left(t\right)\overset{h(m)\&Doppler-FFT}{\to }S_{range-doppler}^{\left[n\right]}\left(t\right)=L^{\left[n\right]}e^{R_{rangle}^{\left[n\right]}\left(t\right),D_{Doppler}^{\left[n\right]}\left(t\right)\left[\frac{4j\pi (f_{c})}{c}\right]} \end{array}$$

In Equations (2) and (3), n∈N, *N* is the number of antennas. h(m) is the Hemming window added to avoid spectrum leakage. R(t) contains the distance information of the target. D(t) contains the velocity information of the target.

**Figure 1:**
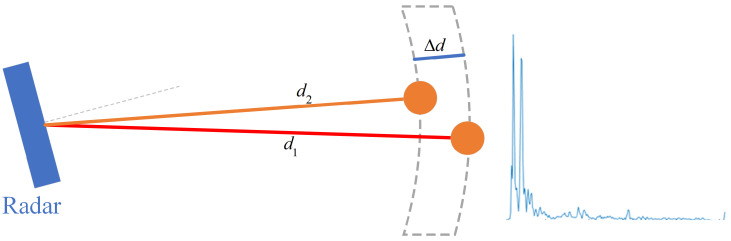
Distance information contained in a Chirp signal.

The object at distance d produces a signal with frequency 2Kd/c, where *K* is the slope of the horizontal and vertical Chirp signal duration $T_{c}$ versus the vertical Chirp signal frequency $f_{Chirp}$ function, and *c* is the speed of light. After the first Range-FFT, we will obtain the information about the target distance. Figure 1 shows the output results. Different objects in space reflect different frequency signals, which the radar uses to distinguish and measure the radial distance to the target. However, according to Nyquist’s sampling theorem, the frequency difference $\Delta f_{IF}$ between two different signal frequencies needs to satisfy certain conditions. If we want to detect two objects, the radial distance difference Δd between the two radar objects needs to satisfy Equation (4). *d* is the radial distance from the radar, $\Delta d=\left|∥d_{n}∥-∥d_{n+1}∥\right|$.

(4) $$\begin{array}{c} \Delta d>d_{res}=\frac{c}{2KT_{c}}=\frac{c}{2B} \end{array}$$

Figure 1 shows that we performed Range-FFT on the first Chirp signal received on one antenna when two objects were in front of the radar. Distance information was obtained for the room’s two targets and other objects.

Similar to the distance resolution, subject to the radar angular resolution, the difference in arrival angle Δθ between two adjacent object antennas in space needs to be greater than the angular resolution $\theta_{res}$ in Equation (5).

(5) $$\begin{array}{c} \Delta \phi >\frac{\lambda }{lN\cos\theta } \end{array}$$

Δϕ is the angular difference between two objects in space, *N* is the number of receiving antennas, *l* is the antenna distance, and λ is the wavelength. However, in Equation (5), there are θ and *l*, which affect the angular resolution. The number of receiving Rx antennas is constant. The angular resolution is highest when the object is directly in front of the radar (θ=90).

**Figure 2:**
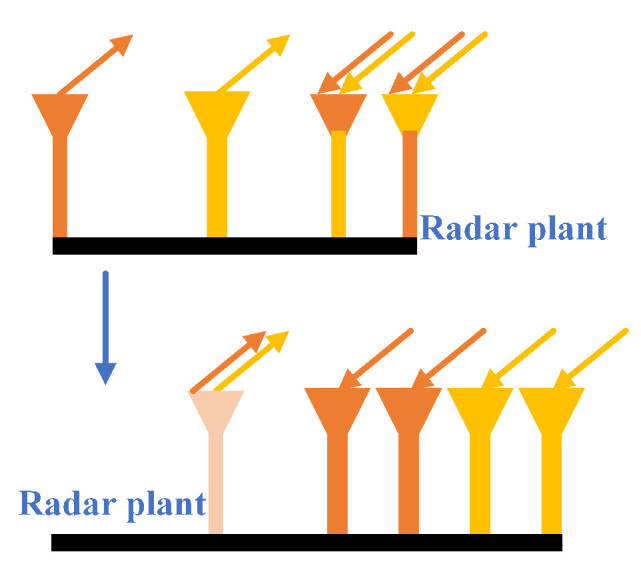
Radar antenna.

In Figure 2, the use of MIMO mode effectively increases the angular resolution of the radar. Since the number of antennas is equal to the number of receiving antennas multiplied by the number of transmitting antennas, according to Equation (5), we know that more antennas mean higher object resolution for this radar.

## 3. Multipath Assisted Localization

### 3.1. MAL Model Framework

**Figure 3:**
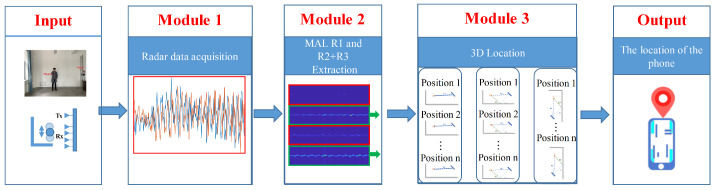
MAL model framework.

Figure 3 describes the overview of MAL: it takes as input the raw signal from 1 RX of the millimeter-wave radar and outputs the 3D position of the phone. To achieve this goal, we designed the following three modules.

- Module 1 is the first step for the MAL system to acquire information about the physical world. In this paper, we use the civilian millimeter-wave IWR1642 Boost development board to send out RF signals and acquire radar IF signals through the DCA1000EVM development board. The signal consists of a real part and an imaginary part. It contains static noise and dynamic targets in space. If we want to obtain the target position, we must perform the next step.
- Module 2 is the basis of the system. Based on module 1, we process the extracted IF signal. It extracts the distance information in the line of sight (LOS) and NLOS environments of the target. To improve the distance resolution of the MAL signal to the target, we first extract all range bins in the original signal and then analyze the Doppler information in each range bin. To avoid the target being interfered with by environmental noise, we obtain the discrete micro-Doppler features at each radial distance of the radar by superimposing the Doppler information in the same range bin for N frames. Finally, by comparing all the micro-Doppler features, the discrete point with the highest energy or the first micro-Doppler feature at a small range distance is identified as the target, and the LOS distance and NLOS distance of the target in the environment are obtained.
- Module 3 is the vital part of the system to obtain the vertical and horizontal position of the target. In this paper, a suitable MAL model is established for the multipath effect. After the input module 1 collects the LOS distance and NLOS distance of the target in the environment, module 2 uses the property of constant radar sensing area, combined with the help of the multipath effect, to calculate the vertical and horizontal distance of the target in space by the MAL model.

Algorithm 1 shows the process of the MAL method. Firstly, we perform the FFT operation in the distance dimension for the data in each frame to obtain the N range bins to be selected. Then, we perform the FFT operation in the velocity dimension for the data in each range bin to obtain the velocity information in different planes. The obtained velocity information is a one-dimensional vector, and by superimposing the velocity information of each frame, we can obtain a two-dimensional $RDM\left(t,j\right)$ matrix. A threshold $Th_{1}$ is set to determine whether there is a target motion feature on the micro-Doppler feature map at this distance, and a threshold $Th_{2}$ is set to determine the direction of the target velocity. When the target is moving radially, the sawtooth pattern is wider on the micro-Doppler feature map, and vice versa, the sawtooth pattern is narrower. By this principle, we can determine whether this distance is a second-order reflection path in the horizontal direction or a second-order reflection path in the vertical direction.
**Algorithm 1:** Steps of MAL
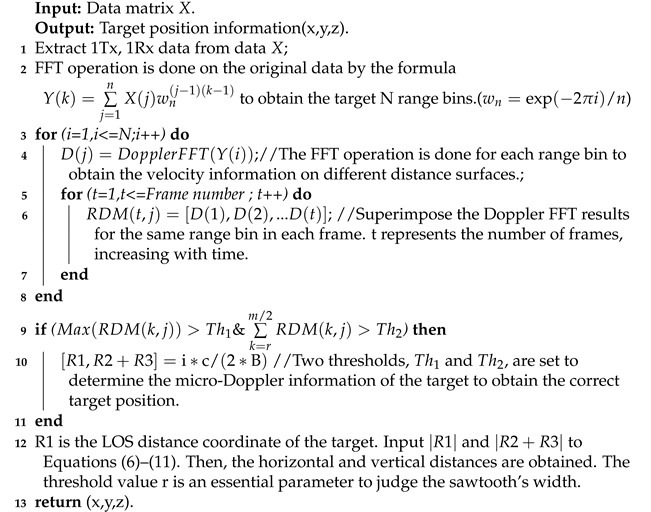


### 3.2. Multipath Effect

Radar signals are affected by various factors in the propagation process. These interferences and influences cause multiple propagation paths of electromagnetic waves. The different paths of signal reflection in space between the target and the radar trigger the different angles and distances of the target received by the radar. According to the literature [16,36], we will define the multi-order signal reflected by the object.

**Figure 4:**
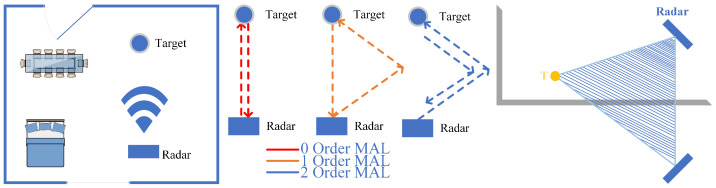
Multipath effect.

- **The zeroth-order MAL:** As the red path in Figure 4 shows, no reflection other than the target occurred during the electromagnetic wave propagation.
- **The first-order MAL:** As the orange path in Figure 4 shows, the electromagnetic wave only experienced a reflection other than the target during the propagation process.
- **The second-order MAL:** As the blue path in Figure 4 shows, the electromagnetic wave first reaches the wall and then returns to the radar through a path other than the red one. It has experienced two reflections other than the target.

The third and above order MAL: Due to the effect of multiple reflections, the artificial analysis path becomes complicated. Furthermore, the increase in the number of reflections leads to the attenuation of the signal energy. This is not discussed in this work. In this paper, based on the defined second and above order MAL model, the shaded part in Figure 4 is again defined as the MAL sensing area. Since no other radar resources are added, the whole MAL sensing area obeys the conservation theorem of the sensing area.

**Figure 5:**
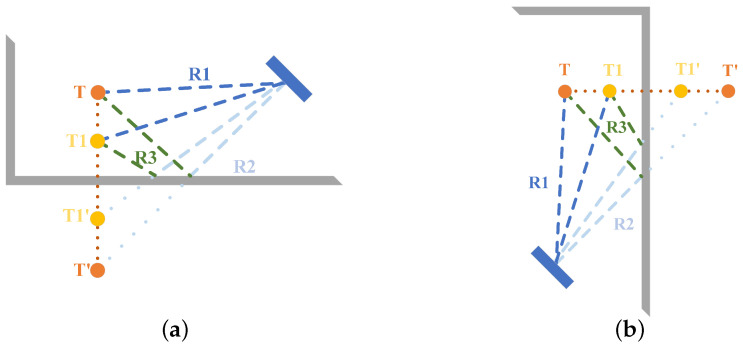
Radar signal reflection paths. (**a**) Side view of the indoor environment. (**b**) Top view of the indoor environment.

Firstly, for the zero-order MAL in Figure 5, the signal goes through R1 twice, and the radar receives the strongest signal strength reflected by the object. This is direct help for our direct object-to-radar radial distance localization.

Secondly, for the first-order MAL in Figure 5, the radar path is either R1→R3→R2 or R2→R3→R1, experiencing a reflection from the ground or wall on the way. In Figure 5, we can see that in the electromagnetic wave of the first propagation path R1→R2→R3, the propagation distance is equal to the second propagation path R2→R3→R1. However, the AoA of the received signal is quite different.

For a common second-order reflection, as in Figure 5, the path of the radar signal is R2→R3→R3→R2. This is common in the MAL model because this path is generally the shortest distance for the second-order reflected signal for the target point, and the received signal energy is generally the strongest.

### 3.3. Acquisition of Target Distance by Micro-Doppler Features

If we want to obtain the position information of the target, how to measure the distance becomes a key step in the whole MAL model. For the LOS path, i.e., the length of |R1|, it is easier to obtain when using the traditional target detection method, but if we want to obtain the distance of |R2+R3| in the NLOS path by such a method, it becomes challenging due to the interference of static noise in the environment. Thus, the key point in the MAL model is obtaining the distance of |R2+R3|. The standard micro-Doppler features generally represent the laws of object motion, and few scholars have used them for distance measurement. The traditional micro-Doppler extraction ignores the fact that there are different motion features on different distance planes. If the motion features on all distance planes are extracted, we can find the phone’s LOS distance and NLOS distance by analyzing the micro-Doppler features on these different distance planes.

**Figure 6:**
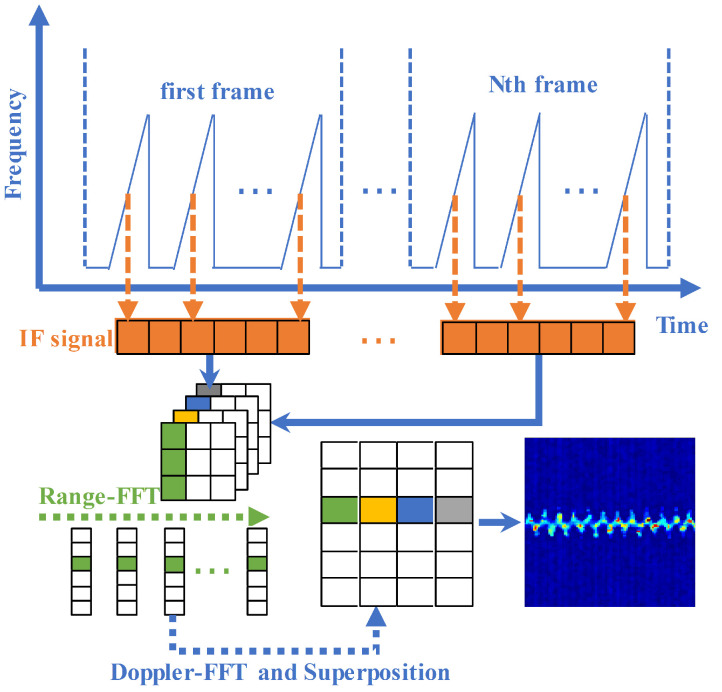
Range–Doppler feature overlay process.

Figure 6 shows the target distance extraction details. We obtained n range bins by taking n-point Range-FFT operation on the IF signal. The process is given in Equation (2), which contains the operation of adding the window function and removing the DC component to suppress the spectrum leakage. Then, the same range bin in all signal frames is subjected to a second Doppler-FFT operation separately. The process is given in Equation (3) to obtain the velocity information in each range bin. Due to the multipath effect, the signal that we acquired contains multiple reflection paths of the target object, and the Doppler information in the second-order MAL reflection model is weak due to multiple reflections, and a single Doppler feature is not enough to distinguish the moving target in space. To extract the required signal reflection path distance and enhance the target object Doppler information in the second-order MAL reflection model, we performed the feature superposition based on the Doppler-FFT operation. We solved the problem wherein the target multi-order reflections are easily overwhelmed by environmental noise by superimposing the velocity information in each range bin.

In this way, we obtain the micro-Doppler features in different distance dimensions, and, finally, the distance of the moving target is obtained by filtering the micro-Doppler information.

**Figure 7:**
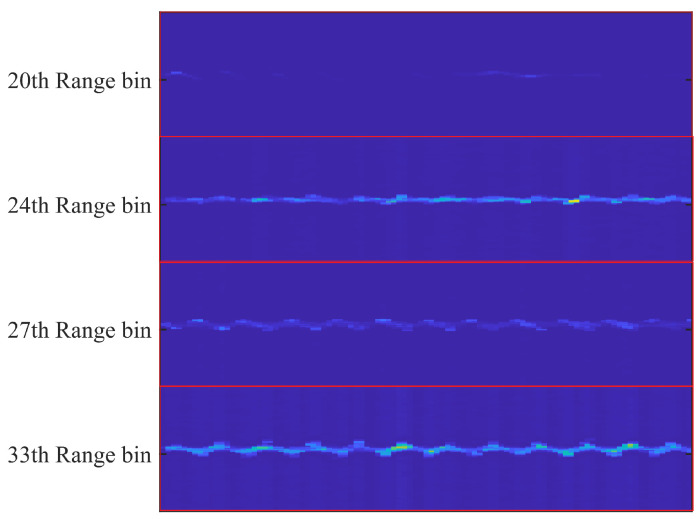
Range–Doppler feature overlay results.

Figure 7 shows the results of the above fabrication. When the volunteers waved two cell phones in the radial direction of the second-order reflections simultaneously, we can see that the MAL model could accurately capture the second-order reflections at two locations with distances of the 24th range bin and 33th range bin. The micro-Doppler information on different range bins has apparent differences.

### 3.4. Horizontal Antenna 3D Location

In this module, the MAL model uses the perceived area constancy principle to estimate the target’s height. First, the model refines the height measurement, and targets of different heights lead to changes in both values of |R1| and |R2+R3|, and the predicted value of $T_{n}$ is obtained using a suitable mathematical relationship equation.

To facilitate the model, we offer the following definitions:

- The volume and wobble amplitude of the rigid target in an actual situation cannot be neglected. However, the MAL model focuses on the central location of the target, so we assume that the target and the radar are considered as one mass point.
- $Radar^{\prime }$ is the mirror image of Radar to the ground, twice the distance from the radar to the ground. $\vec{R2{{}^{\prime }}_{n}}$ is the mirror image of $\vec{R2{}_{n}}$ to the ground.
- The 0th-order reflective linear path distance from the *T* target point to the radar antenna is |R|. The second-order reflective linear path from the *T* target point to the radar antenna is |R2+R3|. The *T* target point projection to radar projection distance is |RDR|. The radar setting height is $H_{n}$. Sn is the area of $\Delta Radar,T,Radar^{\prime }$ by Equation (7), and Pn is half of the perimeter of $\Delta Radar,T,Radar^{\prime }$.

**Figure 8:**
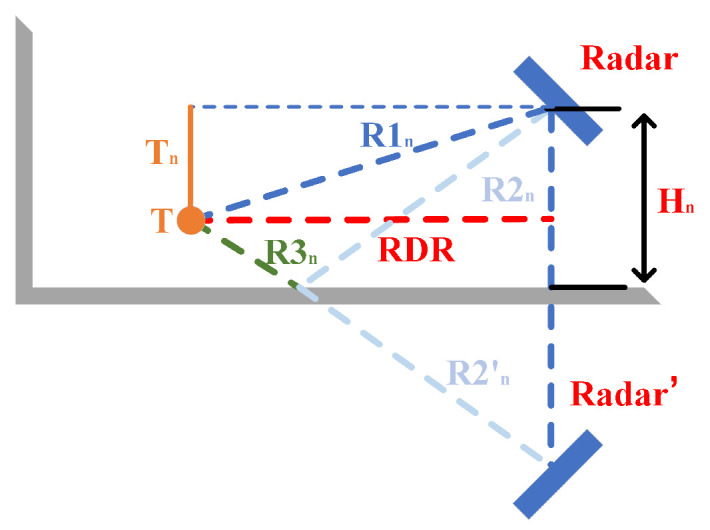
Obtaining the target height.

In Figure 8, the target point height is related to |R1| and |R2+R3| when the target point is at different positions in the vertical direction. When the radar and target projection distance |RDR| is constant, the model yields a constant area no matter how the target point moves. First, we obtain the semiperimeter of the region by Equation (6).

(6) $$P_{n}=\frac{∥R1_{n}∥+∥R2_{n}∥+∥R3_{n}∥+2\cdot ∥H_{n}∥}{2}$$

Subsequently, we obtain the area of the triangle by using Equation (7)

(7) $$S_{n}=\sqrt{P_{n}\left(P_{n}-∥R1_{n}∥\right)\left(P_{n}-∥R2_{n}+R3_{n}∥\right)\left(P_{n}-2\cdot ∥H_{n}∥\right)}$$

According to Figure 8, we find that when the radar is fixed, the area of the target point T ground projection is constant when the projection distance between the target point T ground and the radar ground is the same. Using this property and Equation (8), we derive the projection distance between the ground projection of the target point and the radar ground |RDR|.

(8) $${∥RDR∥}^{*}=\underset{∥RDR∥}{\arg\min}\left[S_{n}-∥RDR∥\cdot ∥H_{n}∥\right]$$

Finally, the target height Tn is obtained by Equation (9).

(9) $$T_{n}=\sqrt{R1_{n}^{2}-{∥RDR∥}^{2}}$$

### 3.5. Single Antenna Horizontal Positioning

**Figure 9:**
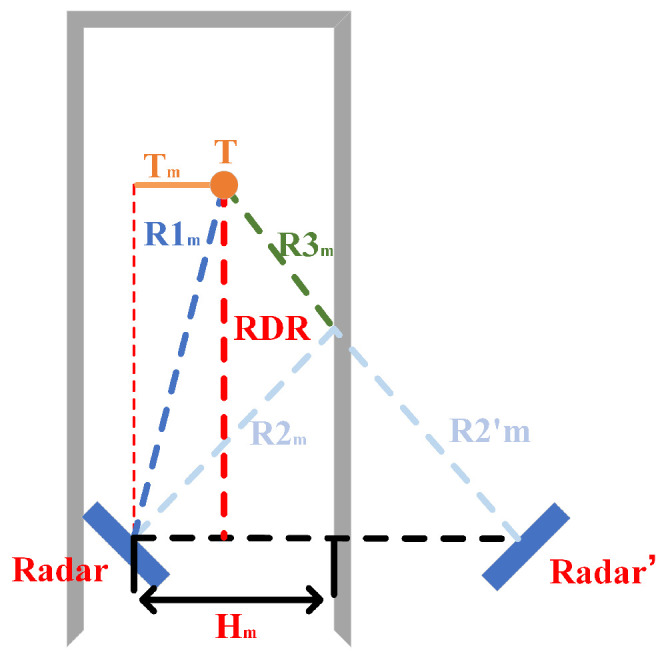
Obtaining the target horizontal axis distance.

For the current MAL model, we have obtained the vector from the target to the radar and the vector of the target in the vertical direction, but the two vectors are not enough to determine the target’s position in 3D space. Thus, we need to obtain the horizontal distance component to the target. As shown in Figure 9, the change in *T* causes the change in the two values of |R1| and |R2+R3|. Similarly, we can locate the target’s horizontal position according to the vertical positioning principle of the horizontal antenna in Section 3.4.

Define $▵Radar,T,Radar^{\prime }$, composed of three points of real radar, virtual radar, and the target point, as the radar sensing area, $S_{n}$ as the area of $▵Radar,T,Radar^{\prime }$, $P_{n}$ as half of the perimeter of $▵Radar,T,Radar^{\prime }$, and $H_{m}$ as the distance from the radar to the wall. According to Equations (6)–(8) and the principle of invariance of the radar sensing area, we can obtain the |RDR| distance, and, subsequently, by Equation (10), the MAL model can obtain the distance of the target from the wall |$T_{m}$|.

(10) $$T_{m}=\sqrt{R1_{m}^{2}-{∥RDR∥}^{2}}$$

**Figure 10:**
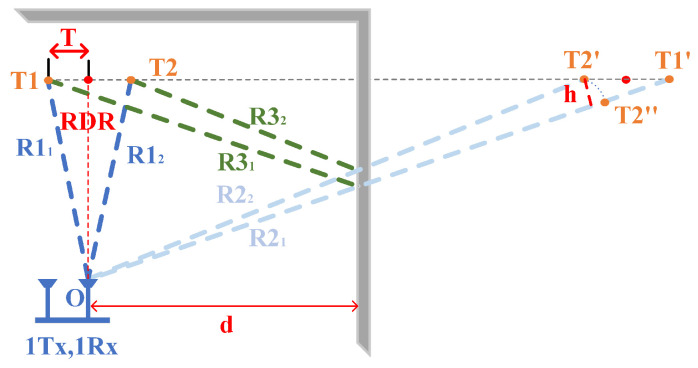
Distinguishing multi-target.

However, it was neglected in the previous sections that when two targets are at a distance less than the radar resolution (two targets are in a range bin), the radar cannot distinguish between the two objects. Thus, we use this one module to achieve the purpose of differentiation of multiple targets. The traditional AOA mode does not consider the reflected noise in the environment, but the “reflected noise” contains much distance to the target. As shown in Figure 10, in this module, we use the radar SISO mode to convert the target orientation angle using distance. The conversion from a polar coordinate system to a Cartesian coordinate system is implemented.

For the IWR1642 Boost, the angular resolution is around 28° in 1Tx,4Rx mode($\theta_{res}=1/\left(N\cos\alpha \right)$).

This module uses information about the constant perceived MAL area. The model discriminates two targets of the same first-order reflection distance, where *P* is $\Delta O,T1,T2^{\prime }$ half of the perimeter and *S* is the area of the $\Delta O,T1,T2^{\prime }$ by Helen’s formula. *T* is the |T1T2| half the size. $||R2_{2}+R3_{2}\left|\right|$ is the second-order reflection path distance of T2. *d* is the distance from the radar to the wall.

(11) $$S=\sqrt{P\left(P-∥R2_{2}+R3_{2}∥\right)\left(P-2\cdot ∥d∥\right)\left(P-∥R1_{1}∥\right)}$$

(12) $${∥RDR∥}^{*}=\underset{∥RDR∥}{\arg\min}\left[S-\frac{1}{2}∥d∥\cdot ∥RDR∥\right]$$

(13) $$T^{*}=\underset{T}{\arg\min}\left(T-\sqrt{{∥R1∥}^{2}-{∥RDR∥}^{2}}\right)$$

Since the MAL model uses the radial distance of the target in the multipath effect to distinguish objects, it is limited by the radar distance resolution, so, in Equation (13), we derive the limit of object resolution for the MAL model. $\Delta O,T2^{\prime },T2^{\prime \prime }$ is an isosceles triangle ($OT2^{\prime }=OT2^{\prime \prime }$) and *h* is the height from point $T2^{\prime }$ to the side of the triangle.

(14) $${\left(\frac{∥RDR∥\cdot ∥T1T2∥}{R2+R3}\right)}^{2}=h^{2}<{∥T1T2∥}^{2}-{∥T2^{\prime \prime }T1^{\prime }∥}^{2}$$

(15) $$\Delta R^{2}<{∥T2^{\prime \prime }T1^{\prime }∥}^{2}<{∥T1T2∥}^{2}\cdot \left(1-\frac{{∥RDR∥}^{2}}{{∥R2+R3∥}^{2}}\right)$$

where ΔR is the distance resolution limit. Theoretically, the MAL model can distinguish between two objects when the condition of Equation (15) is satisfied.

## 4. Experimental Analysis

Since the above theories are presented in an ideal environment, in this section, we integrate the three parts of the MAL model and evaluate the model in four empty room environments to verify the generalization ability of the MAL model. The experiments use the Texas Instruments millimeter-wave radar IWR1642BOOST and DCA1000EVM. Table 1 shows the parameters of the radar. Although the radar data contain four receiving antennas, only one antenna is used in the processing.

**Figure 11:**
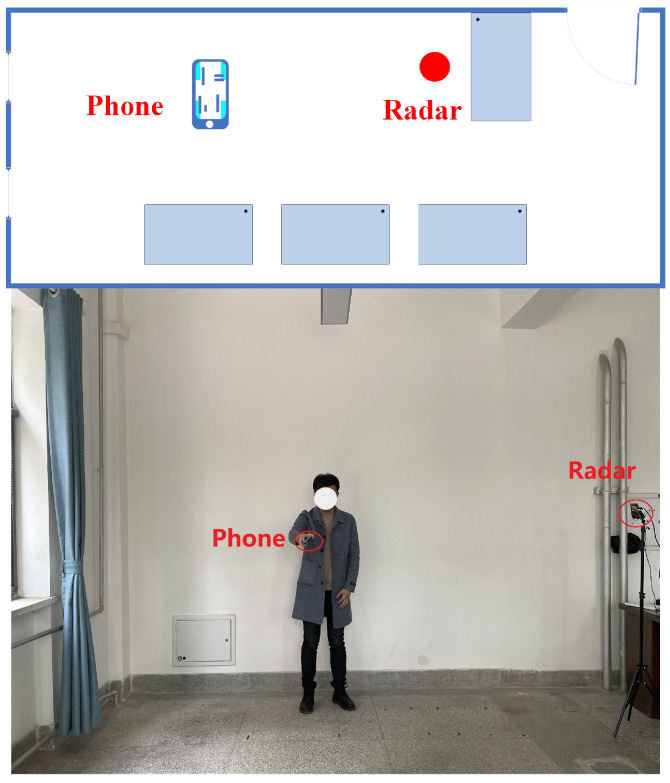
Experimental environment.

Figure 11 shows the floor plan of the empty room with dimensions of 6 m × 5 m. Volunteers shake the phone at a defined position, height, and direction, and during the experiment, volunteers collect 5 sets of data repeatedly at each point. We use the sample mean (Equation (16)) error and standard deviation (Equation (17)) to show the error at each distance point. *S* is the calculation result of the sample standard deviation, η is the average error of the sample, *n* is the number of samples, $x_{i}$ is the error of each measurement, $\bar{x}$ is the average error.

(16) $$\eta =\frac{1}{n}\sum_{i=1}^{n}x_{i}$$

(17) $$S=\sqrt{\frac{\sum_{i=1}^{n}{\left(x_{i}-\bar{x}\right)}^{2}}{n-1}}$$

Since the phone is shaking in the air, the actual positioning position is a range, but to validate the model, the experiment assumes that the center of the phone shaking is the target position. Moreover, at a certain position, to verify the model more comprehensively, we will collect multiple sets of data for different radial velocities of multiple paths.

### 4.1. Localization Error Analysis

Experiments evaluate performance by varying the target-to-radar distance (from 100 cm to 360 cm), target height (from 40 cm to 120 cm), and target-to-reflective wall distance.

#### 4.1.1. Vertical Positioning Error Analysis

**Figure 12:**
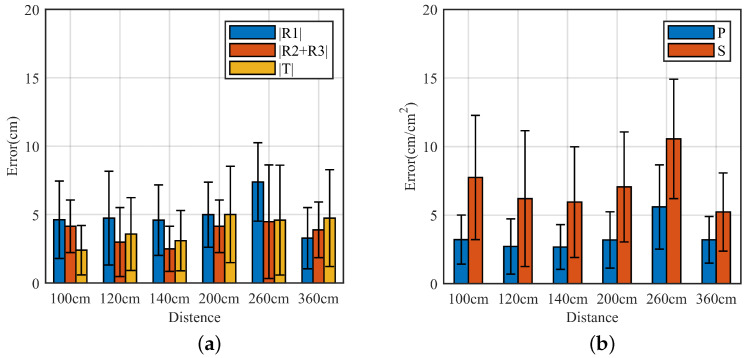
MAL positioning error. (**a**) Vertical distance error. (**b**) MAL model perceived area and half perimeter errors.

In this section of the experiments, the effect of the MAL model in 3D localization is fully verified by setting different heights of the target at different distances. The localization results are shown in Figure 12, where the horizontal coordinates are the different distances, and the vertical coordinates are the height prediction errors. |T| is the prediction result.

Figure 12a shows the MAL model measured |R1|, |R2+R3|, and the target height prediction errors. The MAL sensed area S and half circumference P could be obtained by |R1| and |R2+R3|. We use bar graphs with standard deviations to present the experimental results. The horizontal axis represents the projected distance between the radar and the target, and the vertical axis represents the errors on different heights at different distances. The total MAL model error is within 4 cm on average in the range of 100 cm, and the predicted height is also within 5 cm when the target distance is within 360 cm. Overall, the MAL model error is less affected by the distance between the target and the radar.

Figure 12b shows the error results for the half circumference *P* and the MAL model area *S* measured by the MAL model when the distance is varied. The horizontal axis represents the projected distance between the radar and the target, and the vertical axis represents the error at different altitudes at different distances. The total error of the MAL model half perimeter *P* is within 5.6 cm on average over a range of 360 cm. the total error of the MAL model area *S* is within 10 cm^2^ on average. In the model, the MAL perceived area *S* and half perimeter *P* are essential links to obtain height information, where the errors of MAL perceived area *S* and half perimeter *P* directly affect the localization results. The impact of misjudgment of |R1| range bin and |R2+R3| range bin on height prediction can be minimized by Equation (8).

#### 4.1.2. Horizontal Positioning Error Analysis

**Figure 13:**
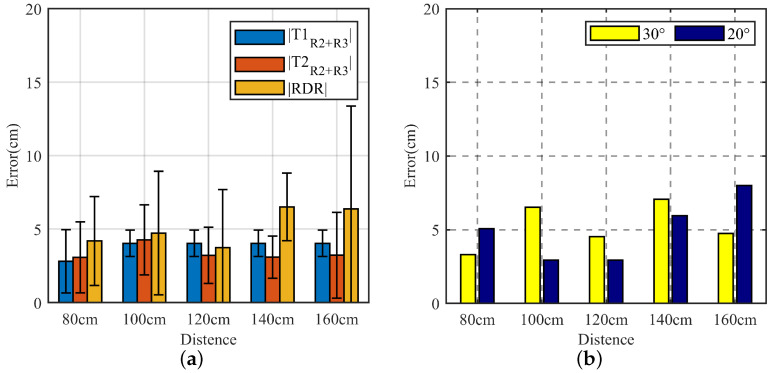
MAL positioning error. (**a**) Horizontal distance error. (**b**) Multi-target resolution errors.

To evaluate the MAL model’s ability to discriminate objects in space, in our experiments, we set two targets directly in front of the radar receiving antenna, and the line between the radar and the midpoint of the two targets was parallel to the wall and 0.5 m apart. We conducted multiple experiments at each distance point and calculated the average error and standard deviation to measure the localization results. According to the descriptions in Figure 10 and Figure 13a,b, the horizontal coordinates are the midpoints of the two target links, different distances to the radar, and the vertical coordinates are the errors at different distances. R1 is the 0th-order reflection distance, $|T_{m}R2|$ is the first distance in the second-order reflection, and $|T_{m}R3|$ is the second distance in the second-order reflection.

In Figure 13a, although the MAL model has explored the information in the multipath effect as much as possible, the indoor reflection is more complex, resulting in similar micro-Doppler features on different range bins, which makes it difficult to identify the exact location, resulting in the maximum standard deviation of the |RDR| predicted distance of approximately 14 cm. However, in the experiments of target differentiation, the probability of such misclassification may be reduced as the target-to-distance increases.

To visualize the advantages of the MAL method in terms of target resolution, we set the object angle (the angle formed by the radar as the endpoint and the rays extending towards each of the two objects) to a constant value. The localization error for the two angle cases is verified. The experiment uses a Cartesian coordinate system to quantify the error. The horizontal axis represents the projected distance between the radar and the target, and the vertical axis represents the horizontal positioning error at different distances. Figure 13b shows the location result.

In the multi-target case, within 1.6 m, the average error of MAL is within 8 cm when localizing multiple targets. As shown in Figure 13b, the increase in the average error of multi-target horizontal localization is insignificant as the distance increases. However, the presence of a large object at 1.6 m leads to an increase in the $|T1_{R2+R3}|$ and $|T2_{R2+R3}|$ measurement error in the second-order reflection, which results in a more significant standard deviation of the final localization. However, the stable mean error shows that the MAL model is feasible in most cases.

#### 4.1.3. Analysis of Positioning Errors in Different Environments

However, in different environments, the distance between the wall and the ground and the radar is entirely different and reflects the signal differently. We collected data in different environments to verify the model’s adaptability to different environments. We analyzed the average and maximum error of MAL model localization in four different environments to verify the performance of the horizontal antenna vertical localization of the MAL model in different environments.

**Figure 14:**
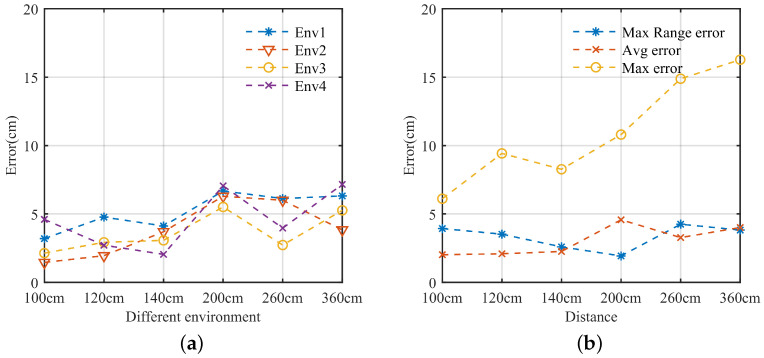
MAL positioning error in different environments. (**a**) Errors in four different environments. (**b**) Maximum error, average error, and difference between maximum and minimum error over the same distance.

After the radar adaptively sets parameters according to different environments, the distance of the target phase from the radar is artificially changed for localization at a vertical distance to test the generalization ability of the MAL model. The four lines in Figure 14a are the average error in the four environments at different distance points. In Figure 14a, we can see that the error variance of the vertical distance is more constant with the distance change in the same environment. In Figure 14a, Max Range error is the distance between the maximum error and the minimum error at the same distance point, Avg error is the average error in different environments, and Max error is the maximum error in the data. In Figure 14b, we can see that the error difference at the same distance in different environments is within 5 cm, which proves that the MAL model is more adaptive in different environments and has excellent generalization ability.

However, in calculating the target height, as the distance increases, the multipath effect may contain distance information from other walls or the ground, leading to the misjudgment of the target range bin, causing the maximum error to keep increasing.

### 4.2. Model Analysis

**Figure 15:**
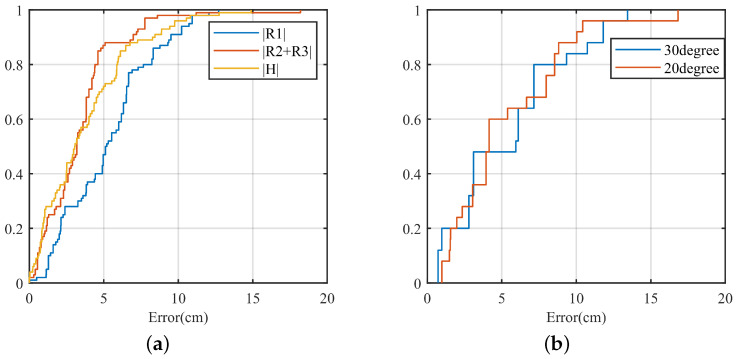
Error accumulation. (**a**) Cumulative error in 3D positioning. (**b**) Multi-target positioning accumulates error.

In this section, we test the MAL’s dual-target lateral position localization error in SISO mode and MAL’s 3D localization error performance, using CDF curves to represent errors ($F_{X}\left(x\right)=P\left(X\leq x\right)$). In Figure 15a, |R1| is the distance error on the target LOS path, |H| is the vertical error of the target, and |L| is the horizontal error of the target. The $\vec{R1}$, $\vec{H}$, and $\vec{L}$ are not parallel to each other in the whole MAL model, and we use these three vectors to represent any point in space. The error results of |R1|, |H|, and |L| are also all within 15 cm.

Figure 15b shows the ability of MAL to discriminate targets in SISO mode. We use the horizontal distance instead of the target arrival angle. In the case of multiple targets, the |R2+R3| range bin of target 1 and the |R2+R3| range bin of target 2 may overlap, and there is interference from other noises, resulting in the misjudgment of the range bin, which causes a larger error.

### 4.3. Comparison Experiment

We compared MAL with two of the more advanced existing mm-Wave radar-based localization methods: the point-cloud-based mm-Wave radar localization method (denoted by Point Cloud [38]), and the conventional mm-Wave radar localization method (denoted by Radar [39]). Both the results of the IWR1642 millimeter-wave radar development board-acquired data were used for comparison.

Compared with point cloud and traditional radar localization methods, the MAL model uses the multipath effect to assist localization. This paper can perform 3D localization when the number of antennas is small, and the analysis is shown in Table 2. Millimeter-wave radar in SISO mode has only one antenna in the vertical direction. Point cloud-based and traditional radar localization methods need the assistance of multiple antennas, so it does not support three-dimensional localization. Compared with other methods, the MAL method still has excellent performance under the limitation of the antenna.

**Figure 16:**
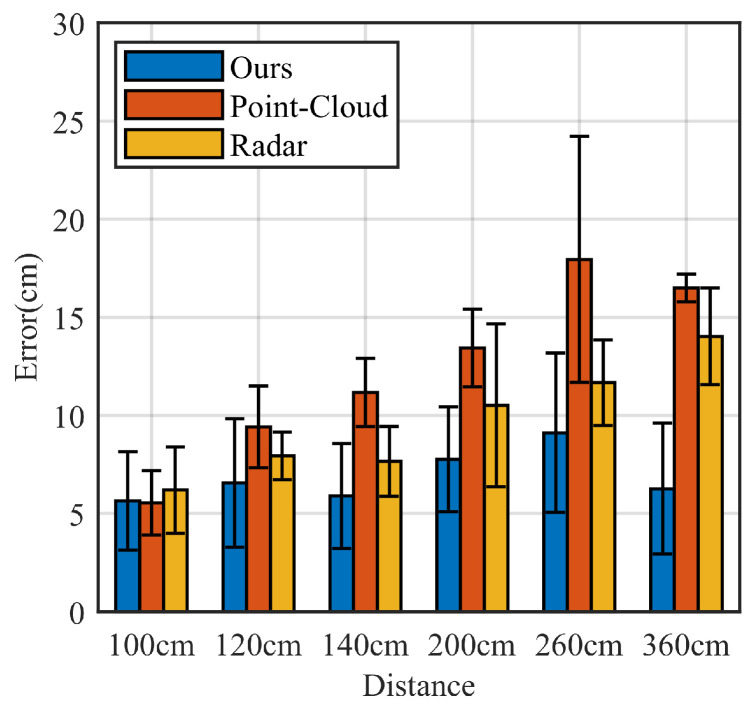
Comparison results of the three positioning methods.

We compared the MAL method with the other methods. Figure 16 shows the comparison results. All three methods have excellent performance for target localization within 100 cm. However, there is no significant increase in MAL error with increasing distance, which is better than the existing methods. The point cloud localization method is based on data clustering to determine the target position by the center point of a large amount of data. The maximum average localization error reaches 17.95 cm. The radar localization method uses the Constant False Alarm Rate Detector method to determine the presence of a target by setting a suitable signal-to-noise ratio threshold, with a maximum average localization error of 14.01 cm. The threshold setting limits its accuracy. In contrast to these two approaches, the MAL uses a dynamic target-sensitive micro-Doppler approach to obtain the target position, with a simple algorithmic process and no need to adjust the appropriate parameters. Moreover, it has a high accuracy rate.

## 5. Discussion

The MAL localization method has high accuracy and privacy and can be used in various scenarios, such as indoor electronic device localization. For the traditional localization method, SISO radar cannot solve the 3D localization problem. In this paper, the principle of invariance of the radar detection area in the MAL model is proposed to extend 1D localization to 3D through the multipath effect. Subsequently, this paper uses distance measurement instead of angle measurement and proves and improves the angular resolution of radar sensing in the MAL model under the antenna limitation. Finally, the performance of the model is evaluated by extensive experiments. The results show that the MAL model solves the 3D localization and angular resolution problems in SISO mode. However, the model still suffers from the following deficiencies.

1. Because of the multipath effect, the model is more environment-dependent. The occlusion on the radar signal propagation path may affect the positioning accuracy when many objects are in a complex indoor environment.
2. Although the MAL model can provide accurate information about the target’s position in 3D space, it cannot track it in real time.

To address the above shortcomings, in future work, we will add point cloud information for the 3D modeling of rooms to improve the adaptability of MAL models to complex environments and to achieve real-time tracking of targets through point cloud-assisted MAL models. The model’s generalization ability can be further improved based on the existing one.

## Figures and Tables

**Table 1:** Radar parameters.

IWR 1642 BOOST	
**Parameters**	**Values**
Carrier Frequency	77 GHz
Number of Transmitting Antennas	1
Number of Receiving Antennas	4
Bandwidth	3997.56 MHz
Single Chrip Signal Duration	60 ηs
Sampling Rate	5000 ksps
Number of Chirps per Frame	128
Number of Frames	150
Number of Samples per Chirp	256

**Table 2:** Comparison of three methods.

Radar Mode	SISO	MIMO
Ours	Support 3D positioning	Support 3D positioning
Point Cloud [38]	Only support 1D positioning	Support 3D positioning
Radar [39]	Only support 1D positioning	Support 3D positioning

## Data Availability

Data is contained within the article.

## Abbreviations

The following abbreviations are used in this manuscript:

MAL	Multipath-Assisted Localization
BDS	BeiDou Navigation Satellite System
GPS	Global Positioning System
GSM	Global System for Mobile Communications
SISO	Single-Input Single-Output
